# Depression is associated with longer emergency department length of stay in acute coronary syndrome patients

**DOI:** 10.1186/1471-227X-12-14

**Published:** 2012-11-05

**Authors:** Donald Edmondson, Jonathan D Newman, Melinda J Chang, Peter Wyer, Karina W Davidson

**Affiliations:** 1 Center for Behavioral Cardiovascular Health, Columbia University Medical Center, New York, USA; 2 School of Nursing, Columbia University Medical Center, New York, USA; 3 Department of Emergency Medicine, Columbia University Medical Center, New York, USA

**Keywords:** Emergency department length of stay, Crowding, Depression

## Abstract

**Background:**

Patient demographic characteristics have been associated with longer emergency department (ED) treatment times, but the influence of psychosocial characteristics has not been assessed. We evaluated whether depression was associated with greater ED length of stay (LOS) in non-ST elevation myocardial infarction (NSTEMI) and unstable angina (UA) patients presenting to a large metropolitan academic medical center.

**Methods:**

We calculated ED LOS for NSTEMI or UA patients enrolled an observational cohort study by taking the difference between ED triage time in the medical record and time of transfer to an inpatient bed from standardized transfer documentation forms. Depression status was defined as current, past, or never by clinical interview and also by self-report on the Beck Depression Inventory.

**Results:**

Participants were 120 NSTEMI/UA patients [mean age= 62, 36% women, 56% Hispanic, 26% Black/African American, 40% NSTEMI, mean global registry of acute cardiac events (GRACE) score= 93.9]. Mean ED LOS was 11.6 hours, SD= 8.3. A multiple linear regression model that included the above demographic and clinical variables, and time of presentation to ED, explained 11% of the variance in ED LOS, F (11, 108)= 2.35, p= .01, R^2^ adj.= .11. Currently depressed patients spent 5.4 more hours (95% CI= .40, 10.4 hours) in the ED on average than patients who had never been depressed.

**Conclusions:**

Currently depressed NSTEMI/UA patients are in the ED for an average of 5 hours longer than those who have never been depressed. Further research is needed to identify the reasons for this difference.

## Background

Thirty percent of acute coronary syndrome [ACS; unstable angina (UA), ST-elevation myocardial infarction (STEMI), and non-ST elevation myocardial infarction (NSTEMI)] patients report substantial depression symptoms during hospitalization, and those patients are at nearly twice the risk of their non-depressed counterparts for ACS recurrence or mortality [1,2]. However, mechanisms for the association between depression and adverse clinical outcome are still in question. The emergency department (ED) is the first point of contact with the medical system for the majority of patients treated for ACS, and recent research suggests that psychosocial factors may influence aspects of the interaction of the patient and emergency care [3,4].

Emergency department length of stay (LOS) is a key marker of ED performance, and longer ED LOS may be associated with adverse clinical outcomes for some conditions [5], in particular those with ACS. Indicators of ED performance have been associated with adverse cardiovascular outcomes in patients who present with chest pain [6], and with worse psychiatric outcomes in ACS patients [7], so we sought to determine whether depressed ACS patients experienced different ED care than non-depressed ACS patients. A number of institutional factors such as hospital occupancy, number of surgical admissions, number of geographically proximate EDs, and number of ED admissions have been associated with longer mean ED LOS at the hospital level [8]. However, patient-level clinical factors such as triage level, utilization of laboratory and diagnostic services, and number of specialty consultations may also influence individual patients’ ED LOS [9]. Though few studies assess individual-level LOS, evidence from the National Hospital Ambulatory and Medical Care Survey suggests that patient demographic characteristics may also be associated with longer individual ED LOS, and one large study found that the presence of a language barrier between physician and patient was associated with significantly longer individual ED LOS [10]. However, the effect of psychological factors on ED LOS has not been described.

We sought to determine whether depression was associated with greater individual patient ED LOS in non-ST elevation myocardial infarction (NSTEMI) and unstable angina (UA) patients presenting to a large metropolitan academic medical center.

## Methods

Participants were ACS patients who had been treated in the New York Presbyterian Hospital (NYP) ED and enrolled in the Prescription Use, Lifestyle, Stress Evaluation (PULSE) study, an ongoing, single site, observational, prospective study of patients with ACS. The primary objectives of the parent study are to identify intermediary phenotypes of depression in ACS patients and the behavioral, biological, and genetic mechanisms that may account for the excess ACS recurrence and mortality risk associated with depression in ACS patients. Patients were eligible to participate if they were diagnosed with acute coronary syndrome, as defined by unstable angina, NSTEMI, or STEMI, and diagnosis was confirmed by 2 independent cardiologists. Patients were ineligible if they were under 18 years old, a prisoner, were deemed unable to comply with the study protocol or had a life expectancy less than 1 year, were not fluent in English or Spanish, or evinced psychosis, bipolar disorder, or personality disorder.

For this substudy, only participants who presented to the NYP ED between February 1, 2009 and June 14, 2010 were included to ensure that data on ED presentation time and transfer time data were accurate. Patients with ST-elevation MI (STEMI) receive rapid percutaneous angioplasty under protocols defined to reduce door-to-balloon times and were excluded from this analysis [11]. Patients with an NSTEMI or UA as per ACC/AHA consensus documents [12] were included in this study.

Serving a racially and socio-economically diverse population, the NYP ED receives over 60,000 visits annually, a large percentage of which are drawn from the predominantly Dominican community surrounding the medical center. The management protocol for patients with chest pain not clearly of non-cardiac origin involves hospitalization following preliminary ED workup for patients not classifiable as having STEMI. The ED does not maintain a chest pain observation unit or provisions for ED stress testing. Participants were enrolled during ACS hospitalization and completed a depression diagnostic interview, depression history, and the Beck Depression Inventory by telephone within 7 days of discharge. ED LOS was calculated retrospectively using data abstracted from participants’ medical records.

### Measures

#### Emergency department length of stay

ED LOS was calculated by abstracting the time of presentation to the CUMC ED, defined as time of ED triage recorded in the medical record, and the time of transfer to an inpatient bed, abstracted from standardized transfer documentation forms, and taking the difference. For patients with LOS longer than 24 hours, admission notes were checked.

#### ED presentation time

ED time-of-day presentation times were trichotomized and analyzed in 3 blocks: 8am-4pm, 4pm-12am, and 12am-8am.

#### Depression

Participants were classified as meeting criteria for current depression (i.e., past two weeks), past depression (i.e., evidence of previous depression episodes, but no current depression), or never having had clinical depression based on results of clinical interview which queried for depression history, as well as lifetime prescription for anti-depressant medications for depression.

We assessed depression using the Diagnostic Interview Schedule-Hamilton interview conducted by a licensed clinical social worker in the 3–7 days post-ACS [13,14]. However, if a participant missed the clinical interview (due to death, rehospitalization, or continuing hospitalization), we used Beck Depression Inventory (BDI) and Patient Health Questionnaire-9 (PHQ-9) scores that participants completed during their initial enrollment during hospitalization, as well as additional screening items added to that hospitalization session about past depressive episodes and lifetime antidepressant medication use in the medical chart, to estimate their past and current clinical depression status.

#### Patient demographic variables

Age, sex, race, and ethnicity were determined by patient self-report. Patient socioeconomic status was quantified using geocoding based on US Census 2000 estimates for the median income of the neighborhood block on which each patient resided at the time of hospitalization. Neighborhood data are superior to self-reported income or education as indicators of socioeconomic status in health research, because neighborhood variables are indicative of both individual socioeconomic status and broader neighborhood characteristics that may influence health [15].

#### Patient clinical variables

Clinical variables were abstracted from participants’ medical records.

##### NSTEMI/UA

ACS diagnosis of NSTEMI or UA was defined by study cardiologists using ACC/AHA research definitions [16].

##### Global registry of acute cardiac events (GRACE) score

The GRACE score is an empirically derived prognostic risk index based on clinical predictors available at the time of an ACS [17]. GRACE variables include age, history of MI or congestive heart failure, heart rate, systolic blood pressure and serum creatinine at hospital presentation, and ST segment deviation, elevated cardiac enzymes, and percutaneous intervention during hospitalization.

This study received ethics approval by the Institutional Review Board (IRB) of Columbia University Medical Center (CUMC; # IRB - AAA9286). All patients were recruited from the clinical departments at NYP. Written informed consent was obtained from all study patients. Completed informed consent documents were then stored in a secure location as per CUMC IRB protocol.

### Statistical analysis

We generated descriptive statistics for all study variables and checked that all variables met the assumptions of linear regression. Participant characteristics were compared across depression status using one-way analysis of variance (ANOVA) for continuous variables and chi-square analysis for categorical variables. To test the primary study hypothesis, we used multiple linear regression to regress ED LOS on age, sex, race, ethnicity, median neighborhood income, ACS type (NSTEMI vs. UA), prognosis (GRACE score), ED presentation time, and depression status. Depression status was analyzed as current depressed vs. past depressed vs. never depressed in order to isolate the potential effect of current depressive presentation on ED LOS from that of more stable depressive personality characteristics that may correspond to any history of depression.

## Results

Of the 139 confirmed UA or NSTEMI patients who were treated in the NYP ED, consented to participate, and were found to be eligible, 120 (86%) had reliable individual-level LOS data, and each had complete data on depression and all other covariates. Thus, participants (Table 1) were 120 NSTEMI/UA patients [mean age= 62, 67% men, 56% Hispanic, 25% Black/African American, 39% NSTEMI, mean GRACE score= 93.5]. Fifteen patients were currently depressed, 28 had been previously depressed but were currently not depressed, and 77 had never been depressed. Mean ED LOS in the sample was 11.64 hours, SD= 8.03.

**Table 1:** Participant characteristics

	**Overall sample characteristics (n= 120)**	**Current depression (n= 15)**	**Past depression (n= 28)**	**Never depressed (n= 77)**
Age, n (SD)	62.3 (11.1)	59.9 (14.8)	60.7 (10.0)	63.1 (10.7)
Male, n (%)*	80 (67)	6 (40)	19 (68)	53 (69)
Black/African American, n (%)**	30 (25)	9 (60)	9 (32)	13 (17)
Hispanic, n (%)	67 (56)	8 (53)	12 (43)	48 (62)
Neighborhood income (SD)	$36,588 ($21,494)	$31,530 ($12,341)	$36,518 ($17,916)	$37,080 ($23,369)
NSTEMI, n (%)	47 (39)	7 (47)	10 (36)	29 (38)
GRACE score (SD)	93.5 (28.3)	94.5 (32.6)	85.9 (27.7)	96.9 (28.7)
Beck Depression Inventory score (SD)**	9.2 (6.9)	15.3 (5.6)	10.6 (7.3)	7.8 (6.5)
ED presentation 8am-4pm, n (%)	62 (52)	6 (38)	15 (54)	41 (53)
ED presentation 4pm-midnight, n (%)*	29 (24)	7 (47)	5 (17)	15 (19)
ED presentation midnight-8am, n (%)	29 (24)	2 (13)	8 (29)	21 (27)
ED LOS (SD)*	11.6 (8.0)	16.3 (11.3)	12.4 (7.5)	10.9 (7.5)

Note: Values represent mean (standard deviation) for each continuous patient characteristic. For categorical variables, n (%) are given.

*p ≤ .05, ** p ≤ .01 for comparison (one-way ANOVA for continuous variables, χ^2^ for categorical variables).

The regression model (Table 2) explained 11% of the variance in ED LOS, F (11, 108)= 2.35, p= .01, R^2^ adj. = .11. Only current depression was a significant predictor of ED LOS, with currently depressed patients spending 5.4 more hours in the ED than patients who had never been depressed (p= .04). Patients who had been depressed in the past were not significantly different than those who had never been depressed (1.34 hours longer for past depression, p= .46). While not statistically significant, participants who presented between midnight and 8am spent 3.03 hours longer in the ED (p= .09) than those who presented from 8am-4pm. We conducted a sensitivity analysis with a log-transformed ED LOS variable, and the results were nearly identical.

**Table 2:** Results of a multiple linear regression model predicting ED LOS from patient demographic and clinical variables, time of ED presentation, and depression status

	**Excess hours of ED LOS (95% CI)**	***β***
Age	−0.07 (−0.26, 0.13)	−0.09
Sex	1.99 (−1.42, 5.40)	0.12
African American race	0.64 (−3.01, 4.30)	0.03
Hispanic ethnicity	2.69 (−0.66, 6.04)	0.16
Median neighborhood income	0.00 (0.00, 0.00)	−0.07
NSTEMI	1.49 (−1.58, 4.57)	0.09
GRACE score	−0.04 (−0.12, 0.03)	−0.16
*Presentation time* (ref= 8am-4pm)		
Midnight—8am	3.03 (−0.52, 6.59)	0.16
4pm—midnight	−2.15 (−5.82, 1.53)	−0.11
*Depression status* (ref= never depressed)		
Current depression*	5.40 (0.40, 10.40)	0.22
Past depression	1.33 (−2.25, 4.91)	0.07

Note: *p < .05; Excess hours of ED LOS associated with a one unit increase in the predictor variable represent the unstandardized B coefficient associated with each predictor.

Though we adjusted for NSTEMI versus UA in the multiple regression models, we also tested whether ED LOS was longer for UA patients than for NSTEMI patients. ED LOS did not differ between the two, UA= 11.0 hours vs. NSTEMI= 12.7; *t* = 1.2, 95% CI for difference= −1.0, 4.6. Further, in a sensitivity analysis, we estimated the same model in 26 STEMI patients from the same cohort. As expected given the defined clinical pathway for STEMI, there was no differential LOS associated with any predictor, including depression [11].

## Discussion

Measures of ED crowding and depression symptoms have both been associated with poor clinical outcomes in cardiac patients [6,18]. While a number of measures of ED crowding assessed at the hospital level, including waiting room census and high-volume hours, were associated with more adverse events in a large sample of chest pain patients, trailing mean ED LOS was not associated with adverse events [6]. Research on whether individual-level ED LOS is associated with poor outcomes in ACS patients is needed. Depression has consistently been associated with adverse outcomes in large prospective cohorts of ACS patients, though a number of candidate mechanisms for this association are still under investigation [1,19].

In this study, we found that clinically depressed ACS patients spent more than 5 hours longer in the ED than patients who had never been depressed. We also found that, as expected, presentation to the ED during the off-peak hours of midnight to 8 am was associated with longer ED LOS. Interestingly, we did not find significant associations between other demographic variables that might be expected to influence ED LOS, such as race, ethnicity, or neighborhood income (a proxy for socioeconomic status), nor did those variables account for the association between depression and ED LOS. There was also no difference in ED LOS between NSTEMI and UA patients, and no association between ED LOS and GRACE score.

While not statistically significant, the data suggest a possible “dose–response” trend between depression and ED LOS. That is, currently depressed participants experienced 5 additional ED LOS hours relative to participants who had never been depressed, while participants who had been depressed in the past experienced slightly more than 1.3 hours longer than those who had never been depressed. Though such a finding should be interpreted as very preliminary, it does give us greater confidence in the primary finding of the study, that depression is associated with longer ED LOS. It also suggests that there may be a more general depressive presentation, as evinced by a history of depression, that may influence patients’ ED experience.

Possible explanations for our findings are that depression may influence how ACS patients present in the ED, their reporting of symptoms, their ability to recruit a family member or friend to accompany and support them, or their interactions with medical staff. At this stage, however, such explanations are speculation, as more research must be conducted. While previous research has shown that posttraumatic stress disorder is associated with longer patient delay to ED presentation in this sample of ACS patients, this is the first study to report an association between depression and ED LOS [4]. We do not yet know why depressed ACS patients are at risk for poor medical outcomes, but delay to medical inpatient services may be one possible factor, of many, contributing to their poor prognosis.

While we found that only depression and time of presentation were associated with individual participants’ LOS, characteristics of each individual and of the specific ED we studied likely influenced our outcome measure. While such influences are obviously multifactorial, they include severity of presenting illness, availability of floor beds, and availability of “spots” on accepting services. Though there may be some variability due to the presence or absence of a private cardiologist or certain clinical conditions, systemic delays in the admission process for patients eligible for inclusion in the parent study (PULSE) are largely administrative in nature and relate to lack of bed capacity or the availability of medical teams. In general, ACS patients are admitted to the Chest Pain Nurse Practitioner service during daytime hours or otherwise to the hospitalist service. Those admitted to cardiology have generally been ruled in for NSTEMI during their stay in the ED or are otherwise complicated. ACS patients are not generally admitted to the CCU unless they qualify for percutaneous coronary intervention (PCI) or are clinically unstable. ACS patients may occasionally be admitted to resident teams other than cardiology. Our knowledge of the admission process, including that through which ACS patients are parsed to floors and provider teams, has not led to a hypothesis that can account for our finding that depressed patients have longer ED LOS.

### Limitations

This study must be interpreted with its limitations in mind. First, these data represent findings from a single, large, urban academic medical center, and as such, the external validity may be limited. Second, the sample size is small, so we may have failed to detect significant associations between other variables and ED LOS; however, since power to detect true associations is limited in small samples, we are convinced of the depression finding. Third, it is difficult to know how our sample may differ from the larger population of all potential (but not confirmed) ACS patients treated in the NYP ED. Since the parent study’s recruitment strategy relies on a confirmation of an ACS diagnosis before approach for consent, our participants may have characteristics that differ from those who do not meet criteria for ACS. The parent study’s participation rate is 85% which gives us confidence that these participants are fairly representative at least of those approached with a confirmed ACS. Fourth, we assessed depression during hospitalization and in the days immediately after using both a self-report screening tool and a clinical diagnostic interview. While our classification of current depression required that participants exhibited evidence of depression prior to ED presentation, we cannot completely rule out the possibility that the experience of increased ED LOS may have influenced recall of depressive symptoms preceding ED presentation. However, we think that such an explanation is unlikely given that many symptoms of depression that are assessed to yield a depression diagnosis are relatively objective (e.g., sleep alterations, changes in eating habits) and therefore not particularly subject to situational or affective recall biases.

Further, we cannot rule out measurement error associated with abstraction of ED LOS from the medical chart. However, if present, measurement error would bias our results away from detecting a difference in ED LOS between depressed and non-depressed ACS patients, as we have no reason to expect this error would be non-randomly distributed between these two groups. Finally, these results are based on a single ED in a large, urban teaching hospital with substantial safety net obligations and a long average ED LOS for ACS patients. As such, it is difficult to know the extent to which these results would generalize to the population of EDs in the United States and around the world. However, we believe these results suggest the need for future research into the possibility that the medical system functions differently for those with psychiatric disorders than for those without, even in acute care for ACS.

## Conclusions

Depressed ACS patients may endure longer ED LOS than non-depressed patients. Given that both ED variables and depression have been associated with poorer post-ACS prognosis, future research should examine factors that may account for the relationship between depression and increased ED LOS for patients classified as ACS, as well as other potential sources of differential medical care for such patients. Further, future research should focus on social and interpersonal factors in the ED that may interact with psychiatric symptoms. It has been hypothesized that the presence of supportive family or friends, and the quality of communication between patients and ED staff, may influence psychiatric outcomes after ACS [20], and they may also moderate the association of depression and ED LOS.

## Competing interests

The author(s) declare that they have no competing interests.

## Authors’ contributions

DE carried out all statistical analyses and drafted the manuscript. JN led data collection and assisted in study conceptualization. MC participated in data collection and cleaning, and participated in hypothesis generation. PW was involved in all stages from conceptualization to interpretation of data analyses, and contributed significantly to writing of all sections of the manuscript. KD is the PI of the parent study, served as senior author and oversaw all steps of manuscript preparation with DE. All authors read and approved the final manuscript.

## Pre-publication history

The pre-publication history for this paper can be accessed here:

http://www.biomedcentral.com/1471-227X/12/14/prepub
